# The role of a chest computed tomography severity score in coronavirus disease 2019 pneumonia

**DOI:** 10.1097/MD.0000000000022433

**Published:** 2020-10-16

**Authors:** Fausto Salaffi, Marina Carotti, Marika Tardella, Alessandra Borgheresi, Andrea Agostini, Davide Minorati, Daniela Marotto, Marco Di Carlo, Massimo Galli, Andrea Giovagnoni, Piercarlo Sarzi-Puttini

**Affiliations:** a Rheumatology Clinic, Università Politecnica delle Marche, Jesi (Ancona); b Dipartimento di Scienze Radiologiche S.O.D. Radiologia Pediatrica e Specialistica, Azienda Ospedaliera Universitaria, Ospedali Riuniti di Ancona, Ancona; c Dipartimento di Radiologia. ASST Fatebenefratelli-Sacco; d Divisione di Reumatologia, Dipartimento di Scienze Biomediche e Cliniche “Luigi Sacco”, ASST Fatebenefratelli-Sacco, Milan University School of Medicine; e Divisione di Malattie Infettive, Dipartimento di Scienze Biomediche e Cliniche “Luigi Sacco”, ASST Fatebenefratelli-Sacco, Milan University School of Medicine, Milan, Italy.

**Keywords:** acute respiratory disease, chest computed tomography, coronavirus disease 2019, outcomes, pneumonia, predictive score, risk factors

## Abstract

The chest computed tomography (CT) characteristics of coronavirus disease 2019 (COVID-19) are important for diagnostic and prognostic purposes. The aim of this study was to investigate chest CT findings in COVID-19 patients in order to determine the optimal cut-off value of a CT severity score that can be considered a potential prognostic indicator of a severe/critical outcome.

The CT findings were evaluated by means of a severity score that included the extent (0–4 grading scale) and nature (0–4 grading scale) of CT abnormalities. The images were evaluated at 3 levels bilaterally. A receiver operating characteristics (ROC) curve was used to identify the optimal score (Youden's index) predicting severe/critical COVID-19.

The study involved 165 COVID-19 patients (131 men [79.4%] and 34 women [20.6%] with a mean age of 61.5 ± 12.5 years), of whom 30 (18.2%) had severe/critical disease and 135 (81.8%) mild/typical disease. The most frequent CT finding was bilateral predominantly subpleural and basilar airspace changes, with more extensive ground-glass opacities than consolidation. CT findings of consolidation, a crazy-paving pattern, linear opacities, air bronchogram, and extrapulmonary lesions correlated with severe/critical COVID-19. The mean CT severity score was 63.95 in the severe/critical group, and 35.62 in the mild/typical group (*P* < .001). ROC curve analysis showed that a CT severity score of 38 predicted the development of severe/critical symptoms.

A CT severity score can help the risk stratification of COVID-19 patients.

## 1 Introduction

A series of cases of unidentified pneumonia with epidemiological links to the Huanan Seafood Wholesale Market were reported in Wuhan, in the Hubei province of China, on December 31, 2019,^[1]^ and it was not long before the Chinese Centre for Disease Control and Prevention identified a novel coronavirus (severe acute respiratory syndrome coronavirus 2 [SARS-CoV-2]) in human epithelial cells as the causative agent.^[2]^ On 30 January 2020, the World Health Organization (WHO) declared the transmission of SARS-CoV-2 the sixth Public Health Emergency of International Concern,^[3]^ and on 22 February 2020, called the disease caused by SARS-CoV-2 coronavirus disease 2019 (COVID-19). On 11 March 2020, the WHO formally announced that COVID-19 was a pandemic.^[4]^ Since 18 March 2020, more than 5.370.375 cases have been confirmed, with 344.454 deaths, which exceeds the number of confirmed cases and deaths associated with SARS and Middle East Respiratory Syndrome.^[5–7]^

Most of the patients have experienced mild symptoms and have made good prognosis, but a minority have developed severe pneumonia, pulmonary edema, acute respiratory distress syndrome (ARDS), or multiple organ failure and died.^[8]^ Mortality among patients critically ill with COVID-19 is substantially high, and the risk of death is increased in older patients with ARDS and co-morbidities such as cardiovascular disease, hypertension, diabetes, chronic pulmonary disease, and cancer.^[9,10]^

Imaging plays an important role in the diagnosis and identification of risk factors for the progression of COVID-19, with chest computed tomography (CT) being considered the first-line imaging modality in highly suspected cases.^[11,12]^

Previous experience with SARS and Middle East respiratory syndrome indicates that CT is more sensitive and specific than X-rays. CT can identify lung abnormalities earlier,^[13]^ and is significantly more sensitive than real-time reverse-transcription polymerase chain reaction in diagnosing COVID-19 (98% vs 71%).^[14]^

The pathologic findings that can be detected at the chest CT scan during COVID-19 are different, among them consolidation, linear opacities, a crazy-paving pattern, bronchial wall thickening, and extra-pulmonary lesions.^[12,15–18]^ The extent and severity of these lesions has a major influence on the prognosis. However, to date, little has been done in trying to attribute a quantitative definition of pulmonary involvement identifiable in CT. Measuring pulmonary involvement of COVID-19 pneumonia quantitatively may help the clinician in defining the severity of the case.

Starting from these considerations, the objective of this study was to quantitatively measure the lung involvement of COVID-19 pneumonia through a CT severity score, and to define a cut-off point of this CT severity score in the definition of severe/critical cases.

## 2 Methods

### 2.1 Study design and participants

Between 20 February and 15 April 2020, data concerning a cohort of patients diagnosed as having COVID-19 pneumonia were retrospectively collected from 3 hospitals in the Italian regions of Lombardy and Marche. The inclusion criteria were:

(1) an epidemiological history;
(2) the real-time reverse-transcription polymerase chain reaction detection of SARS-CoV-2 nucleic acid in throat swabs or the lower respiratory tract, and
(3) at least 1 thin-section CT examination. One the basis of the clinical stages of COVID-19 proposed by WHO,^[19]^

Patients were assigned to 1 of 2 categories: those with mild/moderate disease and those with severe/critical disease. Mild symptomatic patients meeting the case definition for COVID-19 without evidence of viral pneumonia or hypoxia. Moderate patients meeting clinical signs of pneumonia (fever, cough, dyspnoea, fast breathing) but no signs of severe pneumonia, including pulse oximeter oxygen saturation ≥90% on room air. Severe disease was defined a respiratory rate of ≥30 beats per minute, or ≤93% resting oxygen saturation, or arterial oxygen partial pressure (PaO_2_)/fraction of inspired oxygen (FiO_2_) ≤300 mmHg (1 mm Hg = 0.133 kPa), or a ≥50% progression of chest CT findings of pneumonia (fever, cough, dyspnoea, fast breathing) within 24–48 hours.^[20]^ Critical disease was defined as admission to an intensive care unit (ICU) for mechanical ventilation or oxygenation impairment (mild ARDS: 200 mmHg <PaO_2_/FiO_2_ ≤300 mmHg (with positive end-respiratory pressure [PEEP] or continuous positive airway pressure ≥5 cmH_2_O); moderate ARDS: 100 mmHg <PaO_2_/FiO_2_ ≤200 mmHg (with PEEP ≥5 cmH2O); severe ARDS: PaO2/FiO2 ≤100 mm Hg (with PEEP ≥5 cmH_2_O).^[21]^

The patients’ recorded demographic and clinical characteristics included age and sex; the time since symptom onset to hospital admission; co-morbidities (systemic hypertension, diabetes mellitus, heart disease, and chronic obstructive pulmonary disease); symptoms; and clinical and laboratory signs.

All procedures performed in this study were in accordance with the ethical standards of the Institutional Review Board of Luigi Sacco University Hospital and with the 1964 Helsinki declaration and its later amendments or comparable ethical standards. Written informed consent was obtained from all individual participants included in the study.

### 2.2 Image acquisition

The CT examinations used 3 scanners with helical acquisitions in end-inspiration. Dual-energy acquisition on the third-generation dual-source scanner (Somatom Force, Siemens Healthineers, Forcheim) was set at 90/150Sn kV, with modulated mA, a rotation time of 0.25 second, a pitch of 1.05, and a collimation of 2 × 192 × 0.6 mm. The images were reconstructed using iterative reconstructions (Advanced Modeled Iterative Reconstruction, ADMIRE strength 4) at a blending ratio of 0.7: the lung images had a sharp kernel (Bl64) and slice thickness/spacing (ST/SP) of 1.5/1 mm; the mediastinal images had a soft kernel (Br40) and an ST/SP of 3/1.5 mm. The acquisition protocol of the Revolution CT (GE Healthcare, Milwaukee, WI), was set at 120 kV, with modulated mA, a noise index of 17, a rotation time of 0.28 second, a pitch of 0.992, and a collimation of 128 × 0.625 mm. The images were reconstructed using iterative reconstruction (ASIR-V, 30%): the lung images had the lung kernel and an ST/SP of 1.25/1.25 mm; the mediastinal images had a standard kernel and an ST/SP of 2.5/2.5 mm. The acquisition protocol of the LightSpeed VCT (GE Healthcare, Milwaukee, WI) was set at 120 kV with modulated mA, a noise index of 20, a rotation time of 0.5 s, a pitch of 0.984, and a collimation of 64 × 0.625 mm. The images were reconstructed using iterative reconstruction (ASIR, 30%): the lung images had the lung kernel and an ST/SP of 1.25/1.25 mm; the mediastinal images had a standard kernel and an ST/SP of 2.5/2.5 mm.

### 2.3 Image evaluation

#### 2.3.1 Qualitative image analyses

All of the chest CT examinations were independently evaluated by 2 radiologists with respectively 7 (AB) and 20 (MC) years of experience in interpreting chest CT images when blinded to clinical or laboratory data. When there was a difference of opinion, a third radiologist with 30 years of experience (AG) was consulted. Inter-observer variation was calculated using a sample of 25 patients. The images were analysed on 1 of 2 PACS workstations (IDS7 [Sectra] and Centricity PACS [GE Healthcare, Milwaukee, WI]), and viewed using lung (width, 1500 HU; level, −700 HU) and mediastinal settings (width, 350 HU; level, 40 HU). The 2 readers analysed the axial images (but were free to evaluate the multiplanar reformats) for the following features based on the Fleischner Society nomenclature recommendations and similar studies: ground-glass opacities (GGO); consolidation; nodules or fibrosis; the number of lobes with GGO or consolidation; the site(s) of the lesions (subpleural or not); the halo sign, crazy-paving pattern, air bronchogram, of vascular dilatation; interlobular septal thickening; bronchodilatation; pleural thickening, pleural or pericardial effusion; lymphadenopathy and pneumothorax.^[22]^ A sub-pleural lesion was defined as a lesion located no more than 1 cm from the pleural surface. GGO was defined as subtle GGOs seen around the small airways and vessel. The halo sign was defined as GGO around a nodule or mass. Consolidation and GGO were defined as hazy areas of increased attenuation respectively with and without obscuring the underlying vasculature. GGOs with superimposed inter- and intra-lobular septal thickening were defined as crazy-paving, and nodular opacities were defined as round focal solid opacities or GGOs with a diameter of <3 cm. Reticulation was defined as inter- and intra-lobular septal thickening. Sub-pleural bands were defined as thin linear opacities peripheral and parallel to the pleura. Traction bronchiectasis was defined as irregular or distorted dilated airways seen in areas of fibrosis, with an increased bronchus diameter in comparison with the associated pulmonary artery. Vascular enlargement was defined as increased vessel diameter in comparison with a vessel of similar cross section. Lymphadenectasis was defined as lymph nodes with a short axis of >1 cm. The abnormalities were characterised as unilateral or bilateral, and their distribution was categorized as focal (a single focus of abnormality), multifocal (more than 1 focus), or diffuse (involving most of the volume of 1 lung).

#### 2.3.2 Quantitative image analyses

**Figure 1:**
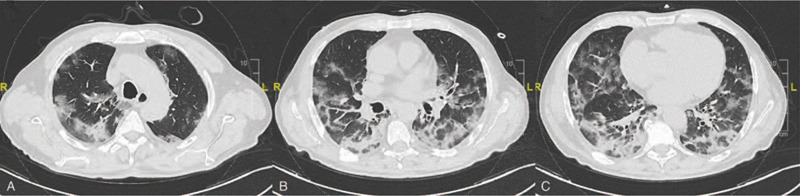
Exemplificative computed tomography scoring system on images of a 68 years old man with severe/critical disease. The total severity score is 53. It was calculated as: for upper level (A), right side (severity score: 9): 3 [mixed consolidation and GGO/crazy paving] × 3 [50–74% distribution] + left side (severity score: 2): 2 [GGO/crazy paving] × 1 [1–24% distribution]; for middle level (B), right side (severity score: 9): 3 [mixed consolidation and GGO/crazy paving] × 3 [50–74% distribution] + left side (severity score: 9): 3 [mixed consolidation and GGO/crazy paving] × 3 [50–74% distribution]; for lower level (C), right side (severity score: 12): 3 [mixed consolidation and GGO/crazy paving] × 4 [≥75% distribution] + left side (severity score: 12): 3 [mixed consolidation and GGO/crazy paving] × 4 [≥75% distribution]. GGO = ground glass opacity.

To assess the severity of pulmonary parenchymal involvement, we attempted to quantify the extent and nature of the abnormalities by scoring each area of lung involvement shown in the axial CT images on the basis of the method described by Ooi *et al.* for SARS.^[23]^ Each lung was evaluated at 3 levels: the upper level (above the carina), the middle level (below the carina up to the upper limit of the inferior pulmonary vein), and the lower level (below the inferior pulmonary vein). The right and left lung were evaluated separately and the results were summed to give the final score for each level. The nature of the lung abnormalities in each area was defined on the basis of a 4-point scoring system detailed, and the percentage of lung involvement at each level was categorized as 0 for normal lung; 1 for <25% lung abnormalities; 2 for 25% to 49% abnormalities; 3 for 50% to 74% abnormalities and 4 for ≥75% abnormalities. The 2 scores (the extent and nature of the abnormalities) were multiplied by each other and added to the scores of all six levels (3 levels on each side). A final radiological severity score ranging from 0 to 96 was attributed to parenchymal involvement. The final score was agreed by all 3 radiologists. In Table 1 the calculation of the CT scoring system is detailed, while in Figure 1 an example is proposed.

**Table 1:**
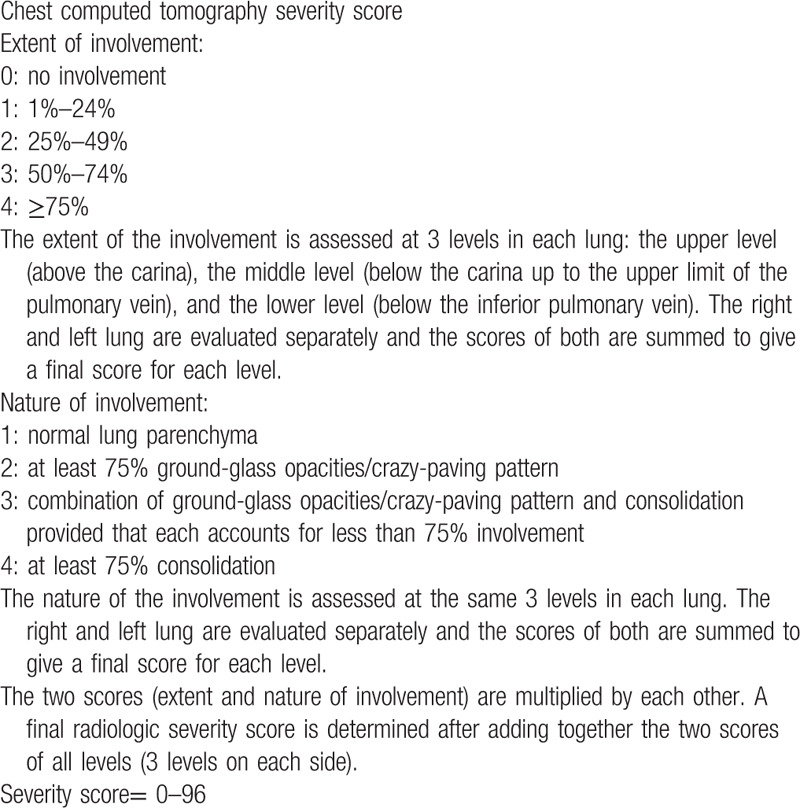
Explanatory table of chest computed tomography severity scoring system.

### 2.4 Statistical analysis

The data were statistically analysed using MedCalc, 64-bit version 19.0.1.0 (MedCalc Software, Mariakerke, Belgium), and are expressed as mean values ± standard deviation or median values and 95% confidence intervals (95% CIs). Categorical variables are expressed as frequencies and percentages, and continuous variables as median values and interquartile ranges. The normality of continuous variables was tested for using Shapiro-Wilk tests. The *χ*^2^ test was used to compare the categorical variables, and the association between the severity scores and the study outcomes (mild/typical *vs* severe/critical) was examined using 1-way analysis of covariance after adjusting for age (the overall p value is that of the main effects of the fixed factor provided by the age-adjusted 1-way analysis of covariance). When significant, pairwise comparisons with Bonferroni's correction were used to ensure an experiment-wide error rate of ≤0.05 and identify the groups with significant differences. The inter-observer agreement concerning the chest CT severity scores was determined by means of intra-class correlation coefficients. A receiver operating characteristics (ROC) analysis was made in order to identify the optimal cut-off severity score (Youden's index) predicting severe/critical COVID-19 pneumonia, and the area under the receiver operating characteristic curve was calculated to quantify its discriminative accuracy.

## 3 Results

### 3.1 Patient characteristics

The study involved 165 COVID-19 pneumonia patients: 131 (79.4%) men and 34 (20.6%) women with a mean age of 61.5 ± 12.5 years. The most frequent symptoms were fever (159 patients, 96.4%), dry cough (85, 51.5%), fatigue (61, 36.9%) and myalgia (44, 26.6%). Sixty-nine percent (95% CI 64–73%) of the patients had at least 1 co-morbidity: hypertension was the most frequent (55/151 patients with available data; 36%, 95% CI 33–41%), followed by cardiovascular disease (37 patients; 24.5%, 95% CI 18–29%), pulmonary disease (29 patients; 19%, 95% CI 15–22%), and diabetes mellitus (26 patients; 17.2%, 95% CI 15.5–20.5%). Only nine patients (5.9%, 95% CI 4–11%]) had a history of hypercholesterolemia.

One hundred and thirty-five patients were classified as having mild/typical disease, and 30 as having severe/critical disease, 21 of whom (70%) were admitted to an ICU (15 directly and 6 during hospitalisation) in which 7 required intubation and 2 died. The severe/critical patients were significantly older (mean age 74.7 ± 12.3 vs 59.9 ± 11.6 years; *P* < .03), had a higher prevalence of diabetes mellitus and cardiovascular diseases, and the median time from disease onset to hospital admission was significantly longer (11 days, range 7–18 versus 7days, range 3–9.8; *P* = .02). There was no difference in the proportion of men and women in the 2 groups (*P* = .088).

### 3.2 CT findings

The most frequent CT finding was multiple lobe involvement (90.9%) with more extensive GGO (86.7%) than consolidation (76.3%). The patients with severe/critical disease had a higher prevalence of consolidation (*P* = .012), a crazy-paving pattern (*P* = .017), interlobular septal thickening (*P* = .001), reticular opacity (*P* = .003), and air bronchogram (*P* = .001), and a higher incidences of lymph node enlargement (*P* = .002), and pericardial and pleural effusion (*P* = .003 for both). Table 2 summarises the CT findings.

**Table 2:**
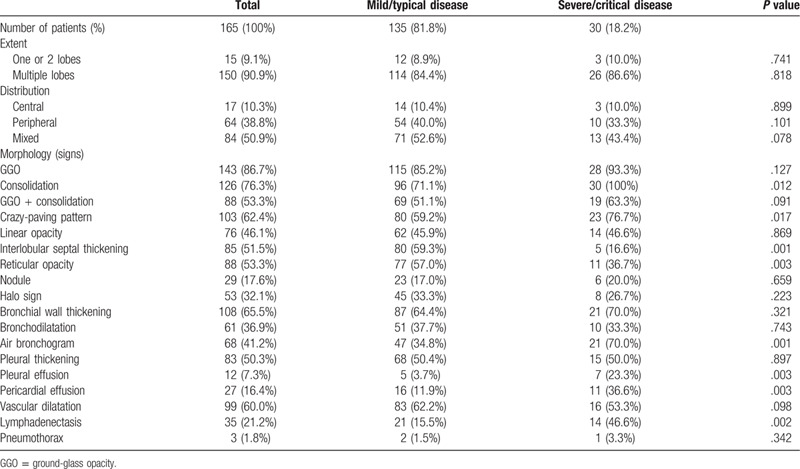
Computed tomography findings in the study population as a whole and the 2 patient groups.

### 3.3 CT severity score

The inter-observer agreement of the 2 readers was excellent (intra-class correlation coefficient 0.908, 95% CI 0.882–0.931; *P* < .001). The mean CT severity score was 63.95 (95% CI 57.30–70.60) in the group with severe/critical disease and 35.62 (95% CI 32.49–38.76) in the group with mild/typical disease (*P* < .001).

**Figure 2:**
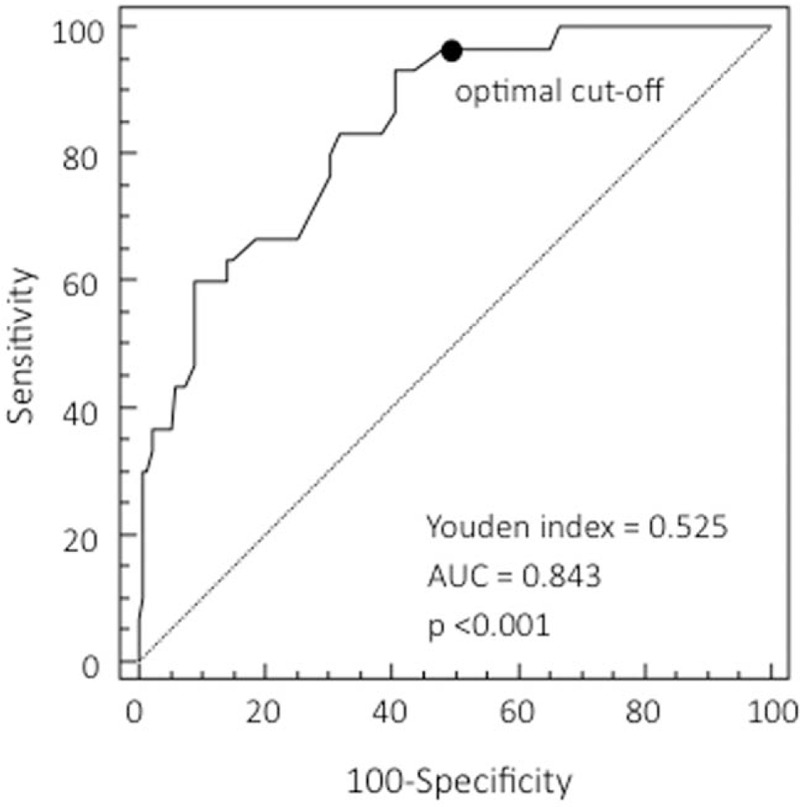
ROC curve showing the prognostic value of the computed tomography severity score. The cut-off value of 38 predicts a severe/critical outcome with 93.3% sensitivity and 59.3% specificity. The area under the ROC curve is 0.843 (95% CI 0.778–0.895), the Youden index 0.525, and provides an index of discriminative performance for severe/critical outcomes. AUC = area under the curve, CI = confidence interval, ROC = receiver operating characteristic.

ROC curve analysis revealed a CT severity score area under the curve (AUC) of 0.843 (95% CI 0.778–0.895; *P* < .0001), and a Youden index of 0.525 (Fig. 2), with an optimal cut-off value of 38 predicting the development of severe/critical symptoms: sensitivity 93.33% (95% CI 77.9–99.2), specificity 59.26% (95% CI 50.5–67.6), and a positive likelihood ratio of 2.29 (95% CI, 1.8–2.90) (Table 3).

**Table 3:**
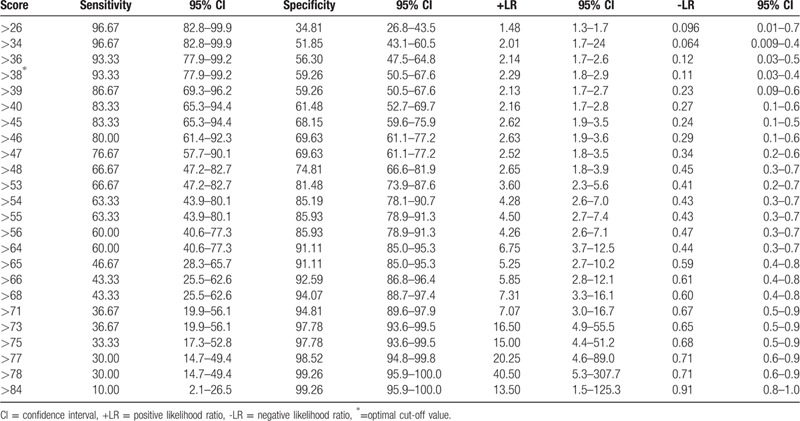
Values and coordinates of the receiver operating characteristic curve.

## 4 Discussion

Patients with severe/critical COVID-19 disease have a poorer prognosis and are at greater risk of dying than patients with mild/typical disease.^[24,25]^

Like those of a number of recent studies, the findings of this study show that CT evidence of consolidation, a crazy-paving pattern, reticular opacity, interlobular septal thickening, air bronchogram, and extra-pulmonary lesions are imaging features of severe/critical COVID-19 pneumonia,^[26–28]^ and their number and frequency make the total whole-lung severity score significantly higher in severe/critical cases. They are particularly evident during the peak progression of the disease (after 1–3 weeks),^[27,29]^ and may be due to further infiltration of the lung parenchyma and interstitium,^[30]^ thus indicating that the virus has invaded the respiratory epithelium, a key target organ in COVID-19,^[31]^ which is characterized by widespread alveolar damage, necrotising bronchitis, and alveoli completely filled with inflammatory exudate.^[32]^

Qualitative indicators alone can distinguish patients with severe/critical or mild/typical disease, but provide little information concerning prognosis.

Li et al have designed a 0–60 scoring system predictive of mortality based on the percentage involvement of each lung and according to the time interval between symptom onset. In patients with an interval between symptom onset and chest CT ≤5 days, they described a CT score of 14.5 (sensitivity 83.3%, specificity 77.3%, adjusted AUC 0.881), while in patients with an interval between 6 and 10 days, a CT score of 27.5 (sensitivity 87.5%, specificity of 70.6%, adjusted AUC 0.895) is predictive of mortality. However, this study only included patients over 60 years of age.^[33]^ Two other studies have described scoring systems measuring the extent of lesions in each lung lobe in order to assess the changes in pulmonary involvement during the course of COVID-19 pneumonia.^[15,16]^ However, the correlation between scores and clinical outcomes was not evaluated. A fourth study used the mean pulmonary inflammation index to assess severity on the basis of the size and distribution of lung abnormalities and found a significant relationship between the pulmonary inflammation index and clinical symptoms, lymphocyte counts and C-reactive protein levels.^[34]^ Our results show that the mean CT score of the patients with severe/critical disease was higher than that of the patients with mild/typical disease, and ROC analysis showed that the optimal cut-off score for predicting a severe/critical outcome was 38, which had a sensitivity of 93.33%, a specificity of 59.26%, and a positive likelihood ratio of 2.29. The identification of this cut-off score should make it easier to interpret CT findings.

This study has a number of limitations. Its retrospective design may have limited its power to identify prognostic factors. Secondly, the critical situation in which the data were collected meant that more detailed information (such as baseline medication use) was not obtained. Thirdly, the 2 groups were not balanced in so far as the group with severe/critical disease was relatively small; more reliable cut-off values will require further studies of larger populations. Fourthly, none of the patients underwent a lung biopsy or autopsy to reflect the histopathological changes. Finally, the specificity of the CT severity score might not be high enough, although we should not overlook the clinical usefulness of CT as an indispensable means of assessing patients with such a highly contagious disease.

## 5 Conclusion

We suggest a chest CT severity score that can be used to evaluate the severity and prognosis of COVID-19 pneumonia, stratify triage patients on the basis of risk, guide treatment choices, and monitor the evolution of this highly contagious and potentially fatal disease.

## Author contributions

**Conceptualization:** Fausto Salaffi, Piercarlo Sarzi-Puttini, Massimo Galli, Andrea Giovagnoni.

**Data curation:** Fausto Salaffi.

**Formal analysis:** Fausto Salaffi.

**Investigation:** Fausto Salaffi, Marina Carotti, Marika Tardella, Alessandra Borgheresi, Andrea Agostini, Davide Minorati, Daniela Marotto, Marco Di Carlo, Massimo Galli, Andrea Giovagnoni, Piercarlo Sarzi-Puttini.

**Project administration:** Fausto Salaffi.

**Supervision:** Fausto Salaffi, Piercarlo Sarzi-Puttini.

**Writing – original draft:** Fausto Salaffi, Marina Carotti.

**Writing – review & editing:** Marika Tardella, Alessandra Borgheresi, Andrea Agostini, Davide Minorati, Daniela Marotto, Marco Di Carlo, Massimo Galli, Andrea Giovagnoni, Piercarlo Sarzi-Puttini.
